# Characterization of CO Adsorbed to Clean and Partially Oxidized Cu(211) and Cu(111)

**DOI:** 10.1021/acs.jpcc.3c05954

**Published:** 2023-12-12

**Authors:** Diyu Zhang, Vladyslav Virchenko, Charlotte Jansen, Joost M. Bakker, Jörg Meyer, Aart W. Kleyn, Irene M. N. Groot, Otto T. Berg, Ludo B. F. Juurlink

**Affiliations:** † Leiden Institute of Chemistry, Leiden University, Einsteinweg 55, 2333 CC Leiden, The Netherlands; ‡ Institute for Molecules and Materials, FELIX Laboratory, Radboud University, Toernooiveld 7, 6525 ED Nijmegen, The Netherlands; § Department of Chemistry and Biochemistry, Fresno State University, 2555 E San Ramon Ave SB70, Fresno, California 93710, United States

## Abstract

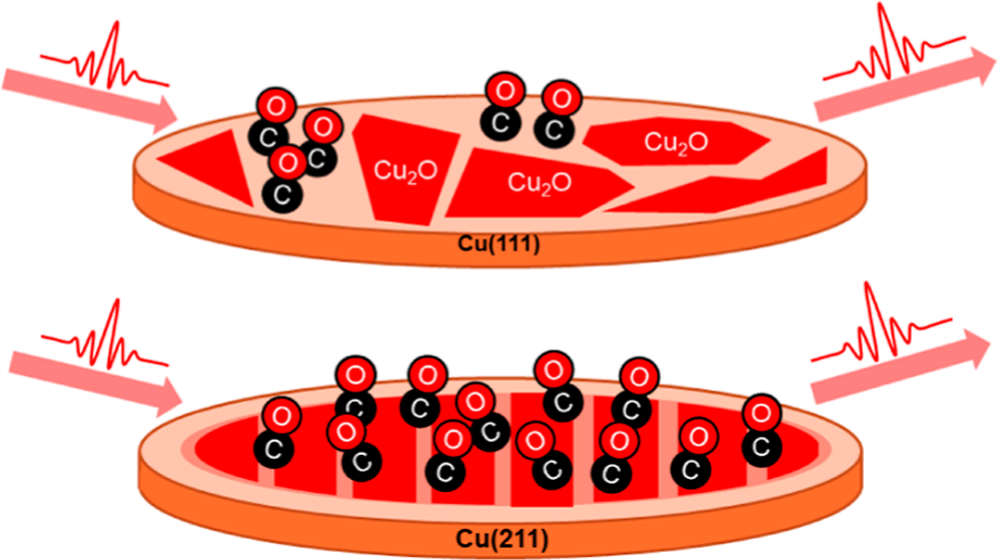

Copper-based catalysts gain activity through the presence of poorly coordinated Cu atoms and incomplete oxidation at the surface. The catalytic mechanisms can in principle be observed by controlled dosing of reactants to single-crystal substrates. However, the interconnected influences of surface defects, partial oxidation, and adsorbate coverage present a large matrix of conditions that have not been fully explored in the literature. We recently characterized oxygen and carbon monoxide coadsorption on Cu(111), a nominally defect-free surface, and now extend our study to the stepped surface Cu(211). Temperature-programmed desorption of CO adsorbed to bare metal surfaces confirms that two sites dominate desorption from a saturated layer: atop terrace atoms of local (111) character and atop step edge atoms with CO bound more strongly to the latter. At low coverage, discrete CO resonances in reflection adsorption infrared spectra can be assigned to these sites: 2077 cm^–1^ for extended (111) terraces, 2093 cm^–1^ for step sites, and additional kink-adsorbed molecules at 2110 cm^–1^. With increasing coverage, in contrast to Cu(111), the infrared spectral features on Cu(211) evolve and shift as a consequence of dipole–dipole coupling between differentially occupied types of sites. Auger electron spectroscopy shows that exposure to background O_2_ oxidizes the (211) surface at a rate nearly 1 order of magnitude greater than (111); we argue that the resulting surface is stoichiometric Cu_2_O, as previously found for Cu(111). This oxide binds CO less strongly than the bare metal and the underlying crystal cut continues to influence the adsorption sites available to CO. On oxidized (111) terraces, broad absorption peaks at 2115–2120 cm^–1^; on oxidized Cu(211), CO adsorbed to step sites appears as a resolved secondary peak at 2144 cm^–1^. This suite of spectroscopic signatures, obtained under carefully controlled conditions, will help to determine the origin and fate of adsorbed species in future studies of reaction mechanisms on copper.

## Introduction

Methanol is synthesized industrially from a mixture of CO_2_, CO, and H_2_ gases over a copper-based catalyst, Cu/ZnO/Al_2_O_3_. The drive to reduce CO_2_ emissions has renewed discussion of the reaction mechanism, in particular the role of various types of sites on the actual industrial catalyst’s surface.^1−6^ Zn and its oxidation to ZnO appear to be of importance in stabilizing intermediates.^4^ Recent reviews summarize the large body of research performed to understand this important industrial process.^7,8^

Some fundamental studies on the mechanism of CO_2_ conversion have employed crystalline Cu as a less complex catalyst, with single-crystal surfaces providing control over the types of available adsorption sites.^4,6,9−12^ Reactions are initiated by exposing a clean and well-ordered Cu surface to reactants from either the background gas or a molecular beam. Surface-bound species are then detected by techniques such as reflection adsorption infrared spectroscopy (RAIRS).^13^

Many experimental and theoretical studies suggest that CO_2_ conversion over Cu is initiated by the formation of formate (HCO_2,ads_) from CO_2_ and a surface-bound hydrogen atom, H_ads_. A direct, Eley–Rideal-like reaction of CO_2_ molecules impinging at the sites of Cu-bound H atoms has been proposed.^12,14,15^ By tracking IR absorption features assigned to formate in the RAIRS spectrum on Cu(111), a reaction probability on the order of 10^–4^ to 10^–3^ was determined for molecular beam-dosed CO_2_ with H_ads_.^12^ Since the probability of CO_2_ direct dissociation on clean Cu is extremely low—reported as 10^–11^ to 10^–9^ on Cu(110)^16^—direct abstraction of adsorbed hydrogen is a plausible first reaction step. The combined IR-molecular beam study claimed that the probability of this direct reaction increases with vibrational and translational energy of the impinging CO_2_.^12^

As discussed in the review by Burghaus, measurement of the extremely low dissociative sticking probabilities for CO_2_ may be confounded by other low-probability processes, such as electron-stimulated dissociation of CO_2_ or mobile subsurface C contamination.^17^ We have recently highlighted similar reasons why the mechanism of CH_3_OH formation over Cu is difficult to determine.^18^ The sticking probability of CO is many orders of magnitude greater than that of CO_2_.^19,20^ Competitive sources of CO could include: residual gas in an ultrahigh vacuum (UHV) chamber; ppb to ppm levels of contamination in the CO_2_ feed gas; and disproportionation of CO_2_ in the high-temperature and high-pressure expansions used to create high-energy supersonic molecular beams. Conventional surface science techniques are as yet unable to distinguish between unavoidable CO contamination (^cont^CO) and CO resulting from CO_2_ (direct) dissociation, (CO_2_ CO).

Furthermore, while a mechanism is an ordered sequence of elementary reaction steps, the formation of complex intermediates may occur via multiple paths. The C atom in HOCO, HCO_2_, CHO, and OCHO may originate from either CO_2_ or CO.^21^ Some studies maintain that, on Cu particles, CH_3_OH formation begins with direct bond breaking and formation of CO_ads_ and O_ads_;^22^ however, isotopic labeling has shown that the carbon source for methanol formation can shift from CO_2_ to CO with temperature.^21^ In order for RAIRS to detect species in a batch-mode reaction, they must persist for a time scale of minutes, so the order of fast reaction steps will be lost.

Contamination by O_2_ is also a source of concern. Although the reactivity of Cu(111) toward dissociation and formation of a copper oxide skin seems mostly limited to defects sites when O_2_ is dosed as a bulb gas, we have recently shown using supersonic molecular beam techniques that reactivity is highly dependent on kinetic energy.^23^ The dissociative sticking probability on the Cu(111) surface rapidly increases to values near unity in the range of 100–400 meV. Such energies are easily obtained for contaminant O_2_ in the supersonic expansions used to create (He-)seeded molecular beams. Hence, even at the common ppm level of O_2_ contamination in the CO_2_ gas feed, gradual buildup of atomic oxygen or oxide patches is to be expected in experiments that expose the catalytic surface for minutes to hours of CO_2_/He-molecular beams. Even the equilibrium between CO_2_ and CO + O_2_ in the gas phase, at temperatures and pressures commonly used for an expansion, may add ^cont^CO and ^cont^O_2_ to the molecular beam.

Concern that ppm-level contamination may influence studies of CO_2_ reactions has motivated our search for a suitable diagnostic method. In particular, we wish to distinguish between ^cont^CO and CO_2_ CO on Cu surfaces that may also be partially oxidized. An expected product of CO_2_ dissociation would be CO adsorbed in association with a site oxidized by the nascent O atom. Computational modeling indicates that this complex will have a distinctively shifted CO vibrational frequency.^24^ Our previous RAIRS study used Cu(111) as the model catalyst surface.^18^ After oxidizing the surface with O_2_ and subsequently adsorbing CO, no unique spectroscopic signature could be assigned to CO associated with sites of oxidation. Indeed, the evidence shows that both the oxide and adsorbed CO grow as separate islands. Oxide patches nucleate on defect sites and develop terrace-inward; CO islands then grow on remaining pristine Cu(111). The intermingling of lone CO molecules with oxidized sites is precluded by this mode of growth.

We now continue this investigation using a surface with a high density of step defects: Cu(211), i.e. Cu[3(111) × (100)] in the van Hove–Somorjai notation.^25^ We expect that the rate of surface oxide formation will be greater than on close-packed Cu(111), because defects lower the O_2_ dissociation barrier.^26^ The greater density of nucleation sites may increase the population of postadsorbed CO that is in direct association with the oxide. In other words, we expect more and smaller oxide patches to be created, and with an increased density of perimeter oxide sites, the density of postadsorbed CO molecules that interact with the oxide will be greater and the infrared spectroscopic signature of these molecules can be identified.

A complication of the stepped Cu(211) surface is that it reconstructs upon exposure to O_2_.^27,28^ The two previous studies on this topic agree that the monatomic steps undergo step doubling. Oxygen atoms are believed to bind to the 4-fold hollow site at the bottom of the doubled {100} micro facets, forming a *c*(2 × 2) overlayer with respect to the Cu(211) surface. The crystallographic plane is preserved by terraces doubling in width, so the Cu surface can be described as Cu(211)(2 × 1) or as Cu[5(111) × 2(100)]. Reconstruction is evident after exposure to more than 0.5 L O_2_ from an effusive source (i.e., a leak valve) followed by annealing to 300 K.^27^ Annealing to higher surface temperatures leads to more complete reconstruction and smoothing of the induced surface corrugation. At lower exposures, but keeping the surface at 300 K, both the original (211) surface and doubled terraces coexist, as observed by scanning tunneling microscopy (STM).^27^ This reconstruction will certainly complicate our interpretation of the coadsorption of CO and O on Cu(211). Finally, we acknowledge the RAIRS studies of CO on clean stepped Cu surfaces performed already in 1975 by Pritchard and co-workers.^29^ However, as noted in an excellent review by Hollins, few (RAIRS-based) studies have characterized CO adsorbed to oxidized Cu single crystal surfaces.^30^ Therefore, the present work aims to systematically characterize CO adsorption to clean and mildly oxidized Cu(211).

## Experimental Section

The samples used here are three Cu single crystals. Two of these are oriented and cut to the (211) plane within ±0.1° accuracy and one to the (111) plane (6N, Surface Preparation Laboratories, Zaandam, The Netherlands). Each sample is laser-welded into a thin, U-shaped high-purity Cu ring. It allows for easy attachment to liquid-nitrogen-cooled cryostats, which are suspended from *x*, *y*, *z*, θ manipulators within our UHV systems. The sample is heated by radiation and/or electron bombardment from an electrically isolated tungsten filament that is held approximately 1 mm behind the crystal. A K-type thermocouple measures the crystal’s (surface) temperature (*T*_surf_). The thermocouple is laser-welded onto the crystal’s edge, between the legs of the U-shaped ring. Repeated cleaning cycles remove contamination from the Cu(211) surface. First, argon ion sputtering is performed at normal incidence (10 min, 500 V, ∼2 μ A, at *T*_surf_ ≈ 400 K). We subsequently anneal at 800 K (10 min). We repeat this procedure at least three times prior to every experiment and check for impurities regularly by auger electron spectroscopy (AES). Experiments are only performed if C, O, and S impurities are below our detection limit. A similar procedure for Cu(111) has been described elsewhere.^18^

The main UHV system used in our studies is a home-built apparatus for RAIRS that has been described in detail elsewhere.^31^ The base pressure of the system is below 2 × 10^–10^ mbar, as measured by an uncalibrated hot cathode nude UHV ion gauge (Varian UHV-24) with a multigauge controller (Varian L8350-301). A Pfeiffer QMA200 quadrupole mass spectrometer (QMS) is used for residual gas analysis. The system is also equipped with a sputter gun (IS40 C1, Henniker Scientific), various leak valves, and either AES optics (ESA100, Staib Instruments) or low energy electron diffraction (LEED) optics (ErLEED, VSI GmbH). The latter share the same port and can only be used sequentially after breaking the vacuum and reestablishing UHV conditions.

The apparatus is equipped to perform RAIRS using an FTIR spectrophotometer (Vertex70, Bruker) with an external liquid-nitrogen-cooled mercury cadmium telluride detector (LN-MCT Mid, Bruker). We generally record RAIRS spectra at 2 cm^–1^ resolution. We continuously supply dry N_2_ gas to the enclosed IR light path. Purge boxes creating the light paths into and out of the UHV chamber are isolated by CaF_2_ windows. We use a grazing angle of incidence of ∼2.5° with the IR light confined to the (01̅1) plane. Hence, the IR light approaches the surface at a right angle with respect to the (100)-type steps of the Cu[3(111) × (100)] surface.

We dose gases using standard leak valves. For dosing O_2_, the gas is introduced with the Cu(211) facing the leak valve at *T*_surf_ = 300 K. There is no directed flow of O_2_ from the leak valve’s orifice. For dosing CO, we use directed dosing. A 6 mm diameter stainless steel tube is attached to a second leak valve’s outlet. Its orifice is aimed at the Cu(211) surface at normal incidence and a distance of approximately 30 mm, which is done at *T*_surf_ = 80 K unless noted otherwise. Doses of CO and O_2_ are reported in units of Langmuir (L).

The TPD spectra and LEED patterns shown in this study have been collected using a second home-built UHV apparatus.^32^ This system also contains LEED and AES optics, an ion gun, leak valves, and pressure measurement systems. The pressure is measured by an uncalibrated cold cathode pressure gauge (IKR 270, Pfeiffer). Here, however, instead of RAIRS capabilities, a QMS (QMA400, Baltzers) is kept in a differentially pumped canister. It monitors the desorption of molecules from the Cu(211) face through a circular orifice at a distance of approximately 1 mm. Repeated positioning of the crystal’s face in front of the differentially pumped canister can be done very accurately (∼10 μm). We use identical cleaning procedures for the same Cu(211) crystal that has been installed in both UHV systems, the only difference being that Ar-ion impact during sputtering is off normal in the latter system.

A third home-built UHV system has also been described previously.^33,34^ It contains a triply differentially pumped supersonic molecular beam that allows for highly controlled impingement of molecules onto the surface of interest. The system’s UHV chamber was recently modified and now allows for RAIRS detection of adsorbates during exposure to the supersonic molecular beam.

The pressure gauges on the three used UHV systems in our laboratory are uncalibrated, and different leak valves do not yield identical dosing. Some gases are dosed more locally onto the surface using 6 mm diameter SS tubes attached to the inside of a leak valve, whereas others (or in another UHV system) are not. Significant differences may occur between the actual exposures and those calculated from recorded pressures. We will repeat this point where it is thought to be of importance.

## Results

**Figure 1:**
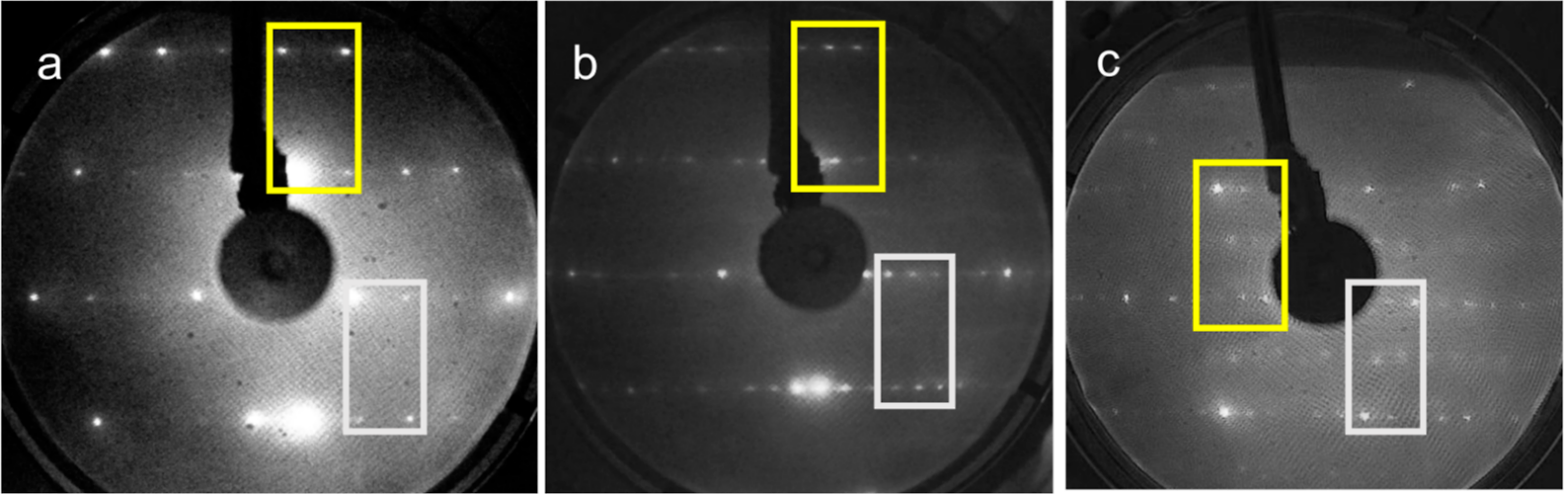
Photographs of LEED patterns at 155 eV and *T*_surf_ = 94 K for (a) cleaned Cu(211), (b) Cu(211) exposed at 100 K to 0.25 L O_2_ and flashed to 450 K, and (c) Cu(211) exposed at 100 K to 4.5 L O_2_, flashed to 450 K. Gray and yellow rectangles encompass examples of changes in patterns as discussed in the text.

Dosing O_2_ onto Cu(211) at *T*_surf_ = 300 K is known to cause dissociative adsorption (see e.g., refs (35)–^36^^37^). However, since the Cu(211) surface reconstructs upon exposure to O_2_, we first compare LEED images of the cleaned Cu(211) surface—before and after exposure to O_2_—with previously published results.^27,28^Figure 1 shows three photographs of LEED patterns. Figure 1a was taken after cleaning Cu(211) and cooling the sample to 94 K. The ratio (spot row spacing)/(spot splitting), as measured from all well-defined spots, has a mean value of 2.21. It is smaller than the theoretical value of 2.45 for the clean fcc(211) type surface.^25^ The observed value would represent a miscut of the crystal’s surface on the order of 2° toward the (311) plane, in which case the average terrace width is slightly less than 3 rows of (111).

The photograph in Figure 1b was taken after exposure to 0.25 L O_2_ at *T*_surf_ = 100 K, flashing to 450 K, and recooling to 94 K. The additional half-order diffraction spots appearing after the exposure to O_2_ are exemplified in the yellow and gray rectangles. We observe them following any exposure greater than 0.1 L O_2_ with a subsequent flash to 450 K. They are in accordance with the (2 × 1) reconstruction. The ratio (spot row spacing)/(spot splitting), as determined again from all available well-defined diffraction spots, increases to 4.69, agreeing approximately with double-high stepped 5(111) terraces.

After dosing 0.6 L or more O_2_, with the subsequent flash to 450 K, we find additional spot rows resulting from a well-ordered lattice of oxygen atoms in the reconstructed steps.^27^ An example is shown in Figure 1c for a 4.5 L O_2_ dose, i.e., the largest dose we have used in our LEED studies. These results are consistent with the (2 × 1) reconstructed Cu(211) surface lattice reported previously. The extent of the reconstruction depends on the exposure, surface temperature, and annealing time.^27^ Under all conditions discussed in the following sections, we dose O_2_ at 300 K and flash to a temperature up to 450 K. The (partial) reconstruction has, therefore, taken place prior to CO exposure at *T*_surf_ < 100 K.

**Figure 2:**
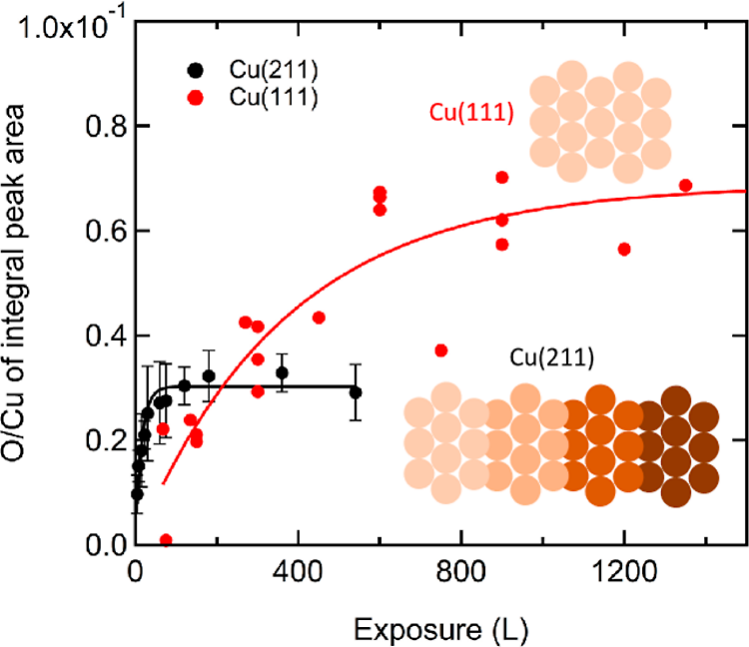
Ratio of oxygen-to-copper integrated AES peak areas as a function of O_2_ exposure. Data acquired for Cu(211) are shown as black symbols and compared to Cu(111) (red symbols). Both data sets are fitted using an appropriate functional form for precursor-mediated adsorption. See text for details. The insets schematically illustrate the two clean Cu surfaces.

We quantify the atomic oxygen coverage by monitoring the AES peaks of oxygen and copper. Figure 2 shows the ratio of integrated AES peaks O/Cu versus the O_2_ dose. Black markers represent Cu(211), and red markers are from our earlier study of Cu(111), where a detailed description of the integration and fitting procedure can be found.^18^ For completeness, we provide in the Supporting Information additional representative AES spectra for the O and Cu regions, obtained after various exposures of the Cu(211) at 300 K (Figure S1).

Two features of the data in Figure 2 catch our attention. First, the rate of increase of the O-to-Cu intensity ratio is much greater for Cu(211) than for Cu(111). After 100 L O_2_ exposure, the ratio has reached its maximum value for Cu(211). For Cu(111) a dose of >1000 L is required to saturate the ratio. The initial steepness of the increase may be taken as a measure of the initial rate of oxidation. We find the ratio of these slopes in the limit of zero dose to be ∼8.5 in favor of Cu(211). This agrees at least qualitatively with previous studies in which Cu(111) was found to exhibit much lower reactivity toward dissociative O_2_ adsorption in comparison with stepped Cu surfaces.^26,27,38^

The second clear difference in Figure 2 is the ultimate O-to-Cu intensity ratio. In our previous study, we compared our results to earlier work on the oxidation of Cu(111) and concluded that a ∼0.069 ratio of AES intensities represents a stoichiometric Cu_2_O layer. For Cu(211), the ratio reaches ∼0.030. Although the rate of dissociation of O_2_ is enhanced by the high step density of the Cu(211) surface, the lowered final ratio suggests that the (211) surface incorporates less oxygen. This interpretation assumes that AES intensities from the different crystallographic planes are similar. The latter is a point of concern, however, and we return to it in the Discussion.

**Figure 3:**
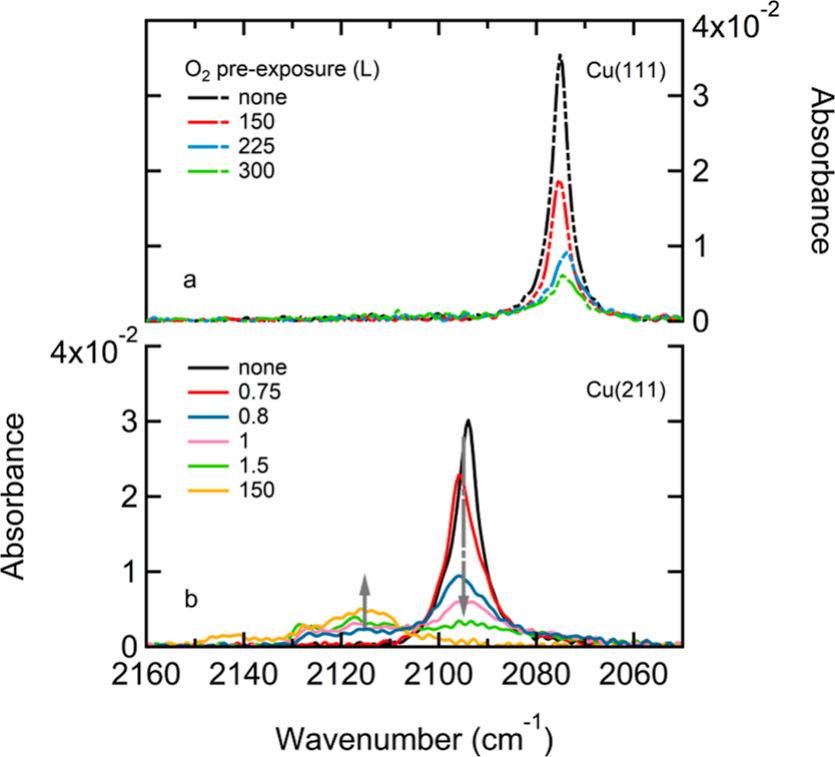
RAIRS spectra of 0.09 L CO on (a) Cu(111) and (b) Cu(211) with various pre-exposures to O_2_. The O_2_ dose is indicated in the legend. For Cu(211), the gray arrows emphasize the simultaneous disappearance of the lower frequency mode and the growth of the higher frequency mode.

Figure 3 shows representative IR absorbance spectra for a fixed CO dose (0.09 L) following various pre-exposures to O_2_ for Cu(111) (upper panel) and Cu(211) (bottom panel). O_2_ was dosed at *T*_surf_ = 300 K. The surface was, subsequently, cooled to ∼90 K, flashed to 450 K, and cooled again to ∼80 K before CO was dosed at. The O_2_ pre-exposure is indicated in the legend. The most prominent differences and similarities are as follows: As a first difference, the internal CO stretch mode at 2075 cm^–1^ for CO/Cu(111) appears at 2095 cm^–1^ for CO/Cu(211). The peak is also clearly broader in the latter case. Both absorptions behave similarly, though, for increasing levels of oxidation. Their intensities drop while center frequencies do not alter significantly. As a third difference, we find an additional broad absorption appearing around 2115 cm^–1^ even when mildly oxidizing Cu(211). It appears already at <1 L exposure to O_2_. This signal increases with oxidation level, but it approaches saturation rather quickly. The CO absorption at 2095 cm^–1^ coexists with this new feature. No comparable band in the spectra of CO/O/Cu(111) appears within the limited oxidation levels shown here. Finally, for Cu(211), we find a weak absorption band near 2143 cm^–1^ for the largest O_2_ exposure.

Examples of fits to the CO absorbance spectra are shown in Figure S2. In Figure S3, we quantify the dependence of these fit parameters on CO exposure, as we have also done for CO/Cu(111).^18^ Our choice of 0.09 L CO to create Figure 3 is based on the collective trends: at this exposure the signal is large, but the qualitative changes seen beyond 0.09 L are absent. In Figure S4 we show the development of the integrated absorbance as a function of CO exposure.

**Figure 4:**
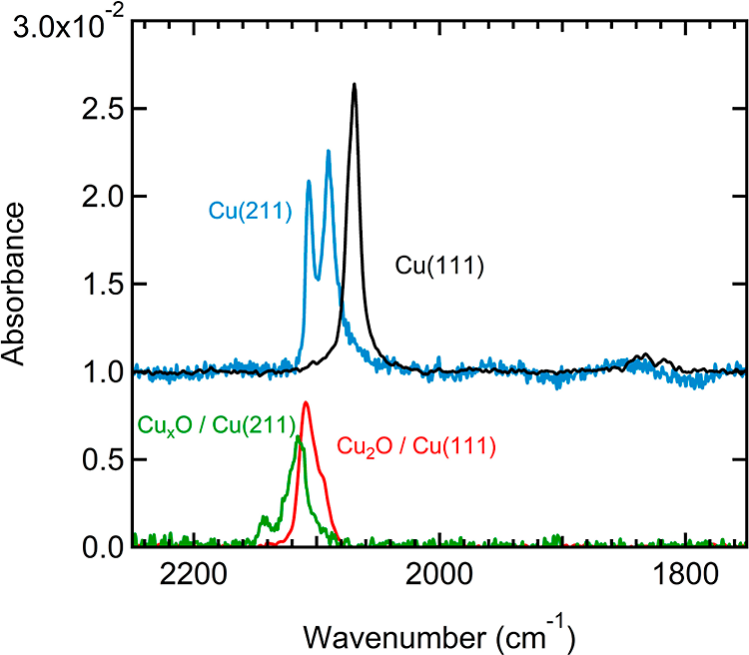
RAIRS spectra of CO on Cu(111) and Cu(211), either metallic (top) or oxidized (bottom) to the maximum level as determined by AES. The subsequent CO dose is ∼1.35 L, i.e., sufficient for CO saturation. The spectra for the metallic surfaces are offset along the absorbance axis by 1% for clarity.

Figure 4 compares IR absorbance spectra of CO at saturation coverage for Cu(111) (black and red traces) and Cu(211) (blue and green traces). At CO saturation (with CO exposure ∼1.35 L), the metallic Cu(111) surface (black trace) shows a single absorption characteristic for atop adsorption at 2069 cm^–1^ and bridge-bound CO with a double absorption near 1833 and 1818 cm^–1^. The metallic Cu(211) surface at CO saturation (blue trace) shows two absorptions in the region associated with atop sites at 2090 and 2106 cm^–1^. We find no clear evidence of bridge-bound CO; signals comparable to the (111) case would not be obscured by the etalon fringes in this frequency region. We also do not find absorbances below 1700 cm^–1^ that would provide evidence for the formation of carboxylates or carbonates.

For the fully oxidized surfaces, there are also clear differences. On Cu(111) (red trace), the absorption is asymmetric with a peak at 2109 cm^–1^ and a shoulder at 2092 cm^–1^. Note that the latter signal only appears for extremely high O_2_ doses and high CO doses. The feature does not show in Figure 3, where surface oxidation was not completed and only a low CO dose was used. For the fully oxidized Cu(211) surface (green trace), we find two well-resolved peaks at 2144 and 2116 cm^–1^. In neither case do we find signals other than those indicative of top-site adsorption, nor do we find absorptions in the range 2150–2240 cm^–1^ that are usually associated with Cu^2+^-bound CO.^39^

**Figure 5:**
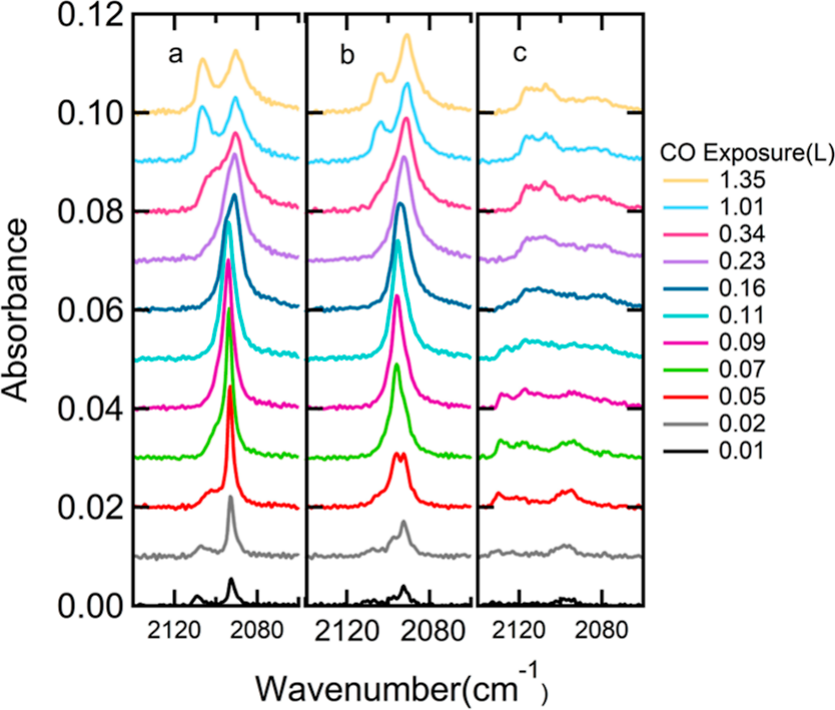
RAIR spectra for varying CO exposures for (a) Cu(211) and after preoxidation by (b) 0.75 L O_2_ and (c) 1.5 L O_2_.

Figure 5 shows RAIRS spectra for increasing CO exposures onto the clean (Figure 5a) and two Cu(211) surfaces with increasing level of oxidation (Figures 5b,c for 0.75 and 1.5 L O_2_ exposure, respectively). For the clean Cu(211) surface, we initially find two resolved peaks at 2093 and 2110 cm^–1^. In the only previous RAIRS study of this system, Pritchard and co-workers also reported two absorptions at 2095 and 2109 cm^–2^.^29^ Only a single signal at 2088 cm^–1^ was found by high-resolution electron energy loss spectroscopy at low CO coverage.^40^ This relatively broad resonance covers the entire frequency region of the doublet observed by infrared absorption.

At intermediate doses, a single absorption is found at 2095 cm^–1^. This single peak was tracked in Figure 3 for increasing preoxidation levels. With increasing CO exposures, this single absorbance blueshifts slightly, as quantified in detail in Figure S3. Finally, it splits into two distinct absorptions at 2090 and 2106 cm^–1^. Pritchard also reported the intermediate appearance of a single absorbance, but at a slightly shifted frequency (2100 cm^–1^) and clearly asymmetric. Also, instead of a final doublet, Pritchard reported a broader single peak at the higher frequency 2110 cm^–1^ that appeared abruptly.

For the lower oxidation level, shown in Figure 5b (predose of 0.75 L O_2_), the behavior is very similar to the clean Cu(211) surface. At this O_2_ exposure, the step-doubling reconstruction of Cu(211) has occurred with oxygen atoms positioned in a *c*(2 × 2) superstructure, as inferred from previous STM studies.^27^ However, the dose is too low to allow for reconstruction of the entire surface, as concluded from Figures 1 and 2. In the IR spectra, the initial absorption at 2110 cm^–1^ does not appear as clearly in comparison to the one at 2093 cm^–1^, but is present. With increasing CO exposure, the lower frequency absorption again becomes dominant, weakly blueshifts, and subsequently redshifts while splitting.

For the doubled initial exposure to O_2_ shown in Figure 5c (1.5 L), the main absorption at 2095 cm^–1^ hardly develops. Instead, weaker and broader features between 2100 and 2140 cm^–1^ appear. With increasing CO exposure, the spectrum develops into the broad band centered at 2115 cm^–1^. Figure S6 quantifies the development of the CO integrated absorbance for a wide range of oxidation levels of Cu(211). In general, the total absorbance at CO saturation is highly nonlinear with the O_2_ exposure.

**Figure 6:**
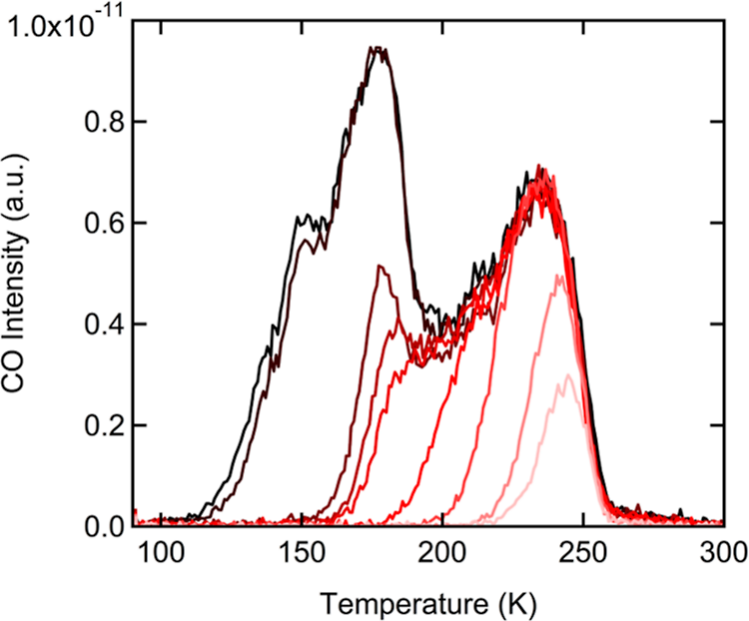
TPD spectra of CO from clean Cu(211) at a ramp rate of 1.5 K/s. CO exposures were increased to (near) saturation, which occurs for this UHV system at 0.23 L. Measurements of doses are incomparable to those mentioned before as a result of varying dosing techniques and pressure measurements. Note that we find a minor but consistent nonlinearity in the temperature ramp causing the fluctuation around 150 K.

Figure 6 shows a series of background-subtracted TPD spectra of CO from Cu(211). Increasing the CO dose is represented by a color variation in the TPD traces shifting from red via brown to black. CO was dosed at *T*_surf_ = 90 K. The heating rate is mostly linear at 1.5 K/s. Unfortunately, a minor nonlinearity always occurs near 150 K and spuriously modulates the signal. The control loop can only be stabilized by modifications that unacceptably limit the accessible temperature range, so we have opted to leave the spectra as shown.

The initial desorption peak appearing at 245 K compares well to results published by Vollmer et al. for Cu(211).^41^ They report the higher temperature desorption feature at 240 K, but did not publish a coverage dependence. For Cu(221), they do report a coverage dependence and show the subsequent developments of a high and lower temperature feature near 225 and 170 K, also very similar to the results for Cu(332).^42^ The higher temperature peak in our data shows overlapping trailing edges. While this is typical for second-order desorption kinetics with no coverage dependence to the desorption energy, we do not expect this to be the case. More likely is a first-order desorption process with a coverage dependence on the desorption energy and/or frequency factor.^43^ The considerably higher temperature of desorption in comparison with Cu(111)^42,44,45^ suggests desorption from step-bound CO, as also concluded by Vollmer et al.^41^ The lower-temperature feature, which peaks around 170 K, initially appears at slightly higher temperatures. These desorption spectra are discussed together with RAIR spectra below.

**Figure 7:**
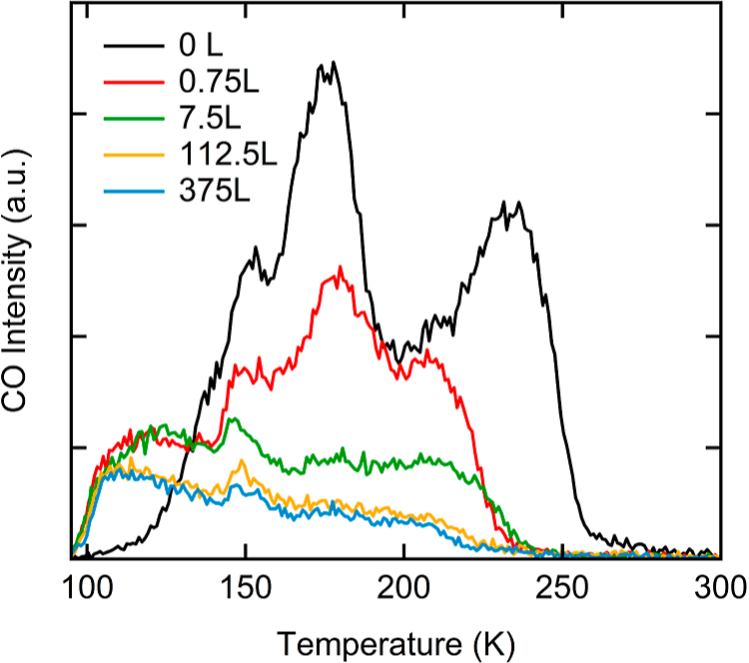
CO TPD spectra from Cu(211) taken at 1.5 K/s with various O_2_ pre-exposures, as indicated in the legend.

Figure 7 shows background-subtracted CO TPD spectra for a range of O_2_ pre-exposures (0 to 375 L). Note that a comparison of absolute doses for CO and O_2_ with earlier figures showing RAIR spectra is improper as we use noncalibrated pressure gauges and different methods of dosing. The CO exposure after the oxidation is fixed at 0.23 L. Although larger CO doses yield variations in the IR spectra (see Figure 5), this dose was established to nearly saturate the desorption of CO from the clean Cu(211) surface from integrated TPD spectra. With increasing pre-exposure to O_2_, both distinct CO desorption peaks from the clean Cu(211) surface decrease. A less intense and broad desorption band stretching from ∼100 to 240 K replaces them. This featureless desorption trace remains present even at very high oxidation levels and constitutes approximately 20% of the CO coverage in comparison with the clean Cu(211) surface exposed to the same CO dose (see also Figure S8). The same characteristic changes in CO desorption from Cu(111) in comparison to the “29” oxide were found before.^46^ Note that the nonlinearity in our temperature ramp around 150 K appears in all spectra.

## Discussion

### Oxidation of Cu(211) by O_2_

We first address the oxygen coverage that we may attain by dosing O_2_ on the Cu(211) surface, including the known step-doubling reconstruction. Witte et al. suggest for the LEED pattern as shown in Figure 1c a coverage of ∼0.08 ML O/Cu.^27^ A *c*(2 × 2) O-overlayer structure of the reconstructed Cu(211)(2 × 1) surface consists of only a single O atom incorporated in every second unit of the doubled (100) facet with a 5-atom row long (111) terrace (see Figure 3 of ref (27)). The coverage equals 1 O atom per 12 Cu surface atoms. They also report an O-to-Cu AES peak-to-peak intensity ratio for the *c*(2 × 2) overlayer that is roughly consistent with this coverage (0.05 ML). However, O_2_ exposures in the earlier study remain limited to 15 L. In our case, 15 L does not saturate the surface: the black (211) markers in Figure 2 only level off after 1 to 2 × 10^2^ L. Lawton et al., using a dome-shaped Cu(111) crystal (d-Cu(111)-10°), report that ∼300 L O_2_ are required to saturate highly stepped surfaces.^26^ Differences may be due to variations in pressure readings from the use of noncalibrated ion gauges, lower step densities available on the d-Cu(111) surface, and also to three-dimensional oxide formation.^27^ Regardless, the dosage at which the O-to-Cu AES intensity ratio saturates in Figure 1 far exceeds that required to observe a LEED pattern consistent with a *c*(2 × 2) oxygen overlayer or to achieve 0.08 ML O/Cu coverage. Hence, the coverage is considerably greater than 0.08 ML.

In Figure 2, the ultimate O-to-Cu AES intensity ratio from O/Cu(111) is approximately double that of O/Cu(211): ∼0.069 vs ∼0.030 from the fitted curves. While this difference may indicate different ratios of O and Cu atoms, it could also be due to Miller-index-specific modulation of AES intensities. These intensities have been documented for Cu surface structures by Armitage and Woodruf.^38^ The variation of specimen current, elastically scattered current, and Auger signals at 62 and 920 eV were measured along the circumference of a cylindrical cyl-Cu[110]^47^ single crystal. No significant difference between the (111) and (211) planes was evident (respectively, 55 and 35° from the (100) reference). Hence, we may assume that the Cu AES intensities in our measurements are effectively identical for Cu(211) and Cu(111). Assuming further that AES intensities for adsorbed oxygen are also independent of the Cu substrate structure, the observed difference in ultimate O-to-Cu intensity must indicate a difference in the saturation coverage of oxygen. Using Cu(111) as a reference, the ultimate O-to-Cu ratio for Cu(211) corresponds to (0.030/0.069) × 0.5 ML = 0.22 ML O/Cu, i.e., approximately Cu_4_O. However, based on the RAIRS signals of postadsorbed CO, we expect the oxygen-saturated surface to be closer to stoichiometric Cu_2_O. This is discussed in full in the Supporting Information.

### CO Adsorption to Cu(211)

One may expect that the sequence in which adsorption sites on clean Cu(211) are populated with CO follow the order of decreasing adsorption energy: kinks and/or defects, step edges (atop and bridged), terraces (atop, bridged, and hollow). In the course of the discussion and with details outlined in the Supporting Information, this expectation will prove true. The documented trend for atop-adsorbed CO to be blue-shifted with increasing binding strength allows us to begin with an interpretation of RAIRS spectra.

The progression in Figure 5a from two resolved peaks at low coverage to a single strong one at intermediate coverage matches the results of Pritchard and Hollins for Cu(211).^29^ We infer that the 2110 cm^–1^ signal at low exposure represents CO molecules strongly bound to a minority of naturally occurring kink sites. The absorption at 2093–2095 cm^–1^ increases with the population of step sites. As the layer becomes more dense, its spectrum is increasingly affected by dipole–dipole coupling (stronger along the step edge direction than normal to it), which shifts the collective resonance to a higher frequency. This interpretation is supported by TPD data as discussed in the Supporting Information in conjunction with Figures S2 and S3.

What physical change in high-coverage layers is responsible for the splitting observed at CO exposures of 0.16 L and above? Two possibilities are attested in the experimental and theoretical literature: population of bridge-bound edge sites as suggested originally in STM studies in the (3 × 1) and (4 × 1) structures,^48^ and population of terraces as suggested by interpretation of TPD spectra.^41^ While the former would introduce another singleton frequency to the layer, its value is known to be ∼200 cm^–1^ lower than the atop-site singleton. No such signal is found in the high-exposure spectra (Figure S4), and it is furthermore too far from the atop resonance to affect vibrational modes in the frequency region of Figure 5. Alternatively, the vibrational frequency of CO molecules adsorbed atop Cu(111) atoms is found in this region. We propose that the final molecules to adsorb are terrace-bound rows that alternate with the preadsorbed edge-bound rows. A general blueshift of the doublet and transfer of oscillator strength to the high-frequency mode is expected and observed. The discussion of Figures S4 and S5 develops additional evidence. Further support could be provided by isotopic dilution measurements and theoretical modeling of this coupling scenario (interdigitated 1D crystals).

The results of our CO TPD experiments are very similar to previous studies of similar systems. Low-Miller-index surfaces only show desorption between 100 and 200 K.^41^ The appearance of a second desorption peak at higher temperatures is generally interpreted as desorption from step sites. Vollmer et al. showed the high-temperature desorption feature to occur between 220 and 250 K for the Cu(211), Cu(221), and Cu(532) surfaces. These stepped surfaces are all described as 3-atom wide (111) planes having various types of step structures. At higher CO exposures, the Cu(221) surface was shown to develop a desorption feature between 100 and 200 K, i.e., overlapping with desorption from the Cu(111) plane. Our TPD spectrum for a CO-saturated Cu(211) surface strongly resembles that of Cu(221), with a major desorption feature peaking near 170 K and the higher feature that is typical for step desorption. The 6-atom-wide (111) terraces of the Cu(332) surface also show a similar spectrum, with a major desorption feature at 170 K and a smaller desorption feature at 230 K.^42^ Here, the relatively small high-temperature peak is consistent with a lower step density and fewer CO molecules desorbing from steps in comparison with Cu(211). Also for Cu(410), consisting of 4-atom wide (100) terraces separated by steps, two desorption peaks occur. The CO desorption feature between 100 and 200 K was assigned to desorption from (100) terrace sites and the desorption feature at 210 K to step desorption.^49,50^

STM studies by Rieder and co-workers also identified CO as adsorbed preferentially to top sites of Cu atoms along step edges of the Cu(211) surface.^51^ Scanning tunneling spectroscopy identified frustrated translational and rotational modes of these molecules,^52^ as previously detected by helium atom scattering,^53^ at 3.0 meV—an energy significantly lower than the 4 meV observed on low-Miller-index Cu surfaces. From the isotropy of the vibration, it was concluded that the shift is caused by a change in bonding character. Because the 5σ orbital is weakly antibonding, a greater 5σ donating character is consistent with a stronger C–Cu bond, hence an increased desorption temperature for binding to steps in comparison with terraces. Strengthening of the internal C≡O bond by the increased donation from the antibonding 5σ orbital would cause a blue shift in the C≡O stretch frequency in comparison with Cu(111). This is also what we find in Figure 5a, where the main initial absorbance is found at 2093 cm^–1^ compared with 2078 cm^–1^ for Cu(111) (see our recent study^18^ and references therein). Even stronger binding to kink sites^54^ shifts the internal frequency further still, to 2010 cm^–1^ for the lowest CO coverages.

In a recent DFT investigation of CO adsorption to Cu(211), Özbek identifies several stable adsorption sites for CO on the narrow (111) terraces.^55^ At all coverages investigated, the energetically most favorable structure involves only step edge sites, with binding energies and vibrational frequencies in line with previous work, but thermal occupancy of terrace sites is not precluded. Three molecular dynamics studies investigate the equilibrium between terrace and step site occupancy using empirical potentials, with a phonon bath and surface relaxation included.^56−58^ The strongly bound step sites are indeed favored, but even at nonsaturated CO coverages a minority of terrace sites is thermally populated. Therefore, exclusive step occupancy and the formation of alternating atop/bridge phases may be a consequence of the lower temperature of STM experiments (30 to 80 K).^48^

Even if step edges were exclusively occupied at the temperature of our RAIRS measurements, we must consider whether the terrace-like TPD signal arises from diffusion to such sites during the temperature ramp. For recombinative H_2_ desorption from faceted Pt(110)(2 × 1), it is known that diffusion may alter the desorption site.^59^ If this were also the case for CO on Cu(211), thermodynamic equilibrium between the step-bound CO “condensed” phase and the CO “gas” on terrace sites would produce (near) zero-order desorption kinetics for the latter: the high-temperature edge of the desorption peak should be independent of initial exposure—as observed, e.g., for desorption of submonolayer coverages of H_2_O from various Pt (covered) surfaces.^60−62^ In contrast, the low-temperature desorption feature in Figure 6 has a clear coverage dependence, its downward shift from 180 to 170 K indicating that both the local coverage and adsorption energy depend on the initial exposure. Hence, we concur with previous TPD studies for CO from stepped Cu surfaces and assign the high-temperature feature to desorption from step edges and the low-temperature feature to desorption from (111) terraces, both being populated by higher CO exposures. This interpretation is supported by the IR absorption signal that appears after comparable exposures, which cannot be assigned to bridge-bound molecules (see Figure S4 and the accompanying discussion for details).

### CO Adsorption to Cu_*x*_O/Cu(211)

The vibrational frequency of adsorbed CO is sensitive to the valence state and coordination environment of surface metal atoms.^39,63,64^ CO on Cu^0^ sites yields absorptions at 2075 cm^–1^ on Cu(111) and at ∼2095 cm^–1^ on Cu(211).^65^ With increasing preoxidation of the same surfaces, these signals decrease and ultimately disappear, as shown in Figure 3 for a relatively small postdose of 0.09 L CO. Only with greater dosing of CO can molecules adsorbed to the oxide be detected. In Figure 4 the typical absorption of CO on Cu^+^ appears between 2100 and 2150 cm^–1^ for both Cu_2_O/Cu(111) and the oxidized surface layer on Cu(211). Evidently, the probability of CO sticking to the oxidized surface is much lower than sticking to clean, metallic Cu(111). Figure S7 shows that the kinetic energy of O_2_ impinging on a 300 K surface does not affect the spectrum of CO subsequently adsorbed to the resulting Cu_2_O/Cu(111) overlayer. In line with the conclusions from our previous study, it is clear that on partially oxidized Cu(111), CO sticks preferentially to metallic Cu sites. Only if there is no Cu^0^ available will it bind, with considerably lower sticking probability, to Cu^+^ sites in the Cu_2_O overlayer. Reaching Cu^0^ sites after impinging onto an oxidized patch of a partially oxidized Cu(111) surface is likely governed by a competition between surface diffusion and desorption.

In contrast to Cu(111), CO adsorbed to a partially oxidized Cu(211) surface can be detected in parallel with CO bound to the bare metal. At the lowest oxidation levels, we find only a decrease in the number of CO molecules bound to Cu^0^ step sites, as represented by the 2093 cm^–1^ absorption in Figure 3. Increasing the oxidation to >0.75 L results in the appearance of CO bound to Cu^+^ as a broad absorption between 2100 to 2150 cm^–1^. The coexistence of oxide- and metal-bound CO is also evident from Figure 5b,c. Under these conditions, the high-temperature CO desorption feature assigned to step-edge adsorption also persists in Figure 7.

With increasing oxidation levels, thus Cu(211)(2 × 1) reconstruction and further O atom incorporation, the step-edge absorption disappears while the broadband assigned to oxide-adsorbed CO grows. TPD spectra show increasingly featureless desorption at lower surface temperatures. The oxidized surface apparently binds less CO, less strongly, than the metal, as evidenced by the decrease in integrated CO thermal desorption with increasing pre-exposure to O_2_ (Figure S8). Accordingly, oxidation also decreases the integrated IR band intensity for all subsequent CO exposures (Figure S6).

Together, these observations suggest that CO molecules impinging on patches of oxidized flat Cu(111) are able to migrate until they reach patches of bare metal. In contrast, when the substrate is stepped Cu(211) some adsorbed CO is trapped onto oxidized Cu patches. The underlying steps apparently promote an oxide morphology that inhibits diffusion, for example, by confining the adsorbate to a single oxidized terrace. That the underlying crystal cut exerts some physical influence over the oxide overlayer is evident from the spectra: For the highest oxidation level (at which both types of crystal are oxidized across the entire surface) and effective CO saturation, the absorption profiles in Figure 3 show similar but not identical distributions of local Cu^+^-type adsorption sites. The low-frequency shoulder for CO on Cu_2_O/Cu(111) is mirrored by a well-resolved peak at a higher frequency for Cu_*x*_O/Cu(211). The dominant feature, however, is only slightly blue-shifted on Cu(211) in comparison with Cu(111). Thus, the oxidation state of exposed Cu atoms is similar in these two cases: approximately Cu_2_O stoichiometry with (mostly) Cu^+^ adsorption sites for CO.

## Conclusions

We have confirmed or newly identified CO adsorption to various types of atop-Cu metallic sites. The (111) terrace, (100) step, and its kink sites are characterized by increasing singleton frequencies observed at 2078, 2093, and 2109 cm^–1^, respectively. Our results strongly suggest that CO adsorbed on mixed Cu^0^ and Cu_*x*_O skins will tend to diffuse and settle at the metallic Cu sites. For CO adsorbed on fully oxidized Cu surfaces, an underlying stepped structure shifts the absorption to higher frequency in comparison with CO adsorbed to atomically flat Cu_2_O overlayers.

On partially oxidized Cu surfaces, the distribution of adsorbed CO molecules among available sites is also sensitive to the underlying crystal facet. Isolated oxide patches on the flat Cu(111) surface do not adsorb a significant fraction of the total adsorbed CO at 90 K, as shown in our previous study.^18^ In this sequel, we have demonstrated that oxide patches on the stepped Cu(211) surface do trap impinging CO molecules at 90 K. (The microscopic basis of this behavior has not been proven, but reduced mobility due to surface texture could explain it.) Therefore, the infrared spectrum of CO on Cu(211) can in principle be affected by the order in which this complex overlayer is prepared. For example, CO generated by dissociative adsorption of CO_2_ to exposed metal is not expected to appear as adsorbed to the oxide, whereas CO dosed from the gas phase can be. We find that CO adsorbed from the gas phase is partitioned between oxidized and nonoxidized areas of the Cu(211) surface with a clear preference for Cu^0^ step edge atoms, as expected from the higher desorption temperature of these sites. The population of metal-bound CO that is perturbed by adjacent oxidized Cu is too low to be detected by infrared absorption. Therefore, the theoretically attested complex of CO_ads_ + O_ads_ on Cu(111), with a distinct internal C≡O stretching frequency, cannot be prepared by the experimental methods employed here. While it may still be created by dissociative adsorption of CO_2_, if the CO product so formed does not appear at a vibrational frequency distinct from those discussed in our results, then the possibility of CO contamination must be ruled out by other means.

## Supporting Information Available

AES quantification methods of oxygen surface concentration, representative RAIRS spectra of CO on Cu(211) including fitting procedures, fit parameter variations with coverage, and temperature dependence, RAIRS CO absorbance dependence on oxygen precoverage, RAIRS CO spectra for Cu(111) dosed by background and molecular beam methods, the dependence of integrated CO TPD spectra on oxygen precoverage, and a comparison of RAIRS spectra for high CO coverage on Cu(211) with results published by others (PDF)
