# Supplementary material for: Characterization of CO Adsorbed to Clean and Partially Oxidized Cu(211) and Cu(111)
Source: J Phys Chem C Nanomater Interfaces. 2023 Dec 12;127(50):24158–67. doi: 10.1021/acs.jpcc.3c05954 (PMC10749469; doi:10.1021/acs.jpcc.3c05954)
Supplement: Supplementary file 1 — jp3c05954_si_001.pdf [file jp3c05954_si_001.pdf]

Characterization of CO Adsorbed to Clean and Partially Oxidized Cu(211) and Cu(111)

Diyu Zhang,† Vladyslav Virchenko,† Charlotte Jansen,† Joost M. Bakker,‡ Jörg Meyer,† Aart W. Kleyn,† Irene M.N. Groot,† Otto T. Berg,¶ and Ludo B. F. Juurlink*,†

†*Leiden Institute of Chemistry, Leiden University, Einsteinweg 55, 2333 CC Leiden, the Netherlands*

‡*Radboud University, Institute for Molecules and Materials, FELIX Laboratory, Toernooiveld 7, 6525 ED Nijmegen, the Netherlands*

¶*Department of Chemistry and Biochemistry, Fresno State University, 2555 E San Ramon Ave SB70, Fresno, CA 93710, USA*

E-mail: l.juurlink@chem.leidenuniv.nl

Phone: (+31) 71 527 4221

Supporting Information Available

AES of O/Cu(211)

The clean Cu(211) surface was exposed to different amounts of O_2 at 300 K. Representative AES spectra for the O and Cu regions are shown in Figure S1. The O_2 exposures are indicated in the legend. All AES data were normalized to the Cu(LMM) peak at 910 eV. The data shown in Figure 2 of the main manuscript are the ratio of the integrated peaks for

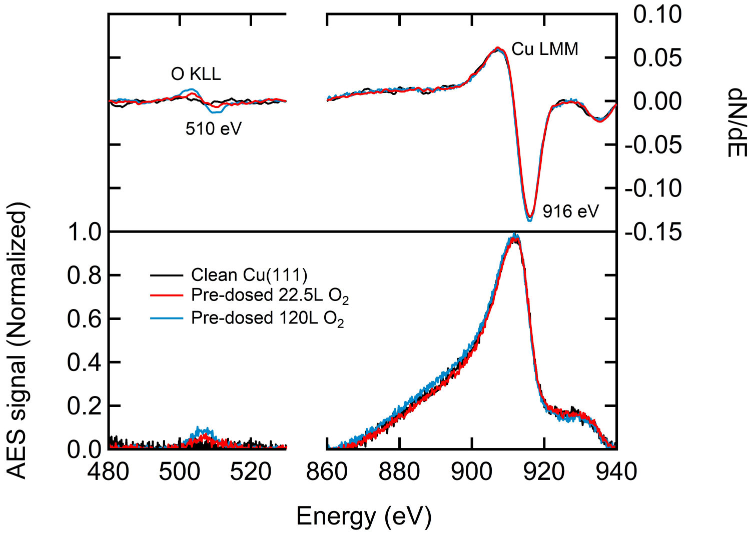

Figure S1: Normalized AES signals (lower panel) and differentiated spectra (upper panel) of the oxygen and Cu regions obtained after different exposures of Cu(211) to O_2.

O and Cu as shown in the bottom panel.

Given that Cu(111) oxidizes as Cu_2O, the halved AES intensity ratio in Figure 2 suggests that the stoichiometry of oxidized Cu(211) is Cu_4O. But the default assumption that AES intensity from O atoms on the surface is the same for Cu(111) and Cu(211) is suspect. The incorporation of O atoms into the Cu(211) surface induces the $c(2 \times 1)$ reconstruction, in which the initial O atoms are thought to be incorporated in the double-high (100)-type step facets.[1,2] These step facets are not oriented along the macroscopic [211] surface normal; their normal is rotated by 35°. Also, the (111) facets in both the unreconstructed and reconstructed (211) surface are rotated by 19° from the macroscopic surface normal, but in the opposite direction from the step facet. Hence, it is likely that Auger electrons from O atoms in the oxidized and (partially) reconstructed Cu(211) surface have angular distributions that peak well away from the surface normal. Since our cylindrical mirror analyzer for Auger electron collection is oriented along the normal direction, the AES signal for O atoms on Cu(211) is expected to be attenuated relative to Cu(111). Therefore, despite the use of the Cu signal as an internal standard, the integrated AES intensity ratio from a flat surface is not a reliable way to infer the stoichiometry of a stepped one.

As an alternative, we consider the internal vibration of adsorbed CO, which is known to be sensitive to the oxidation state of a Cu substrate. The strongest IR absorption band of CO adsorbed to either oxidized surface overlaps in the range 2100 - 2150 cm^{-1}, as shown in Figure 4 of the main manuscript. This evidence points to a similar level of oxidation in both cases, namely Cu_2O stoichiometry with (mostly) Cu^+ adsorption sites for CO. Unique to oxidized Cu(211) is a weak, discrete high-frequency peak at $\sim$2143 cm^{-1}. It may well represent oxidation of the unique double-high (100) step facets of Cu(211), which create a minority of adsorption sites that are not present on the flat oxide.

RAIR spectra fitting and results for CO/Cu(211)

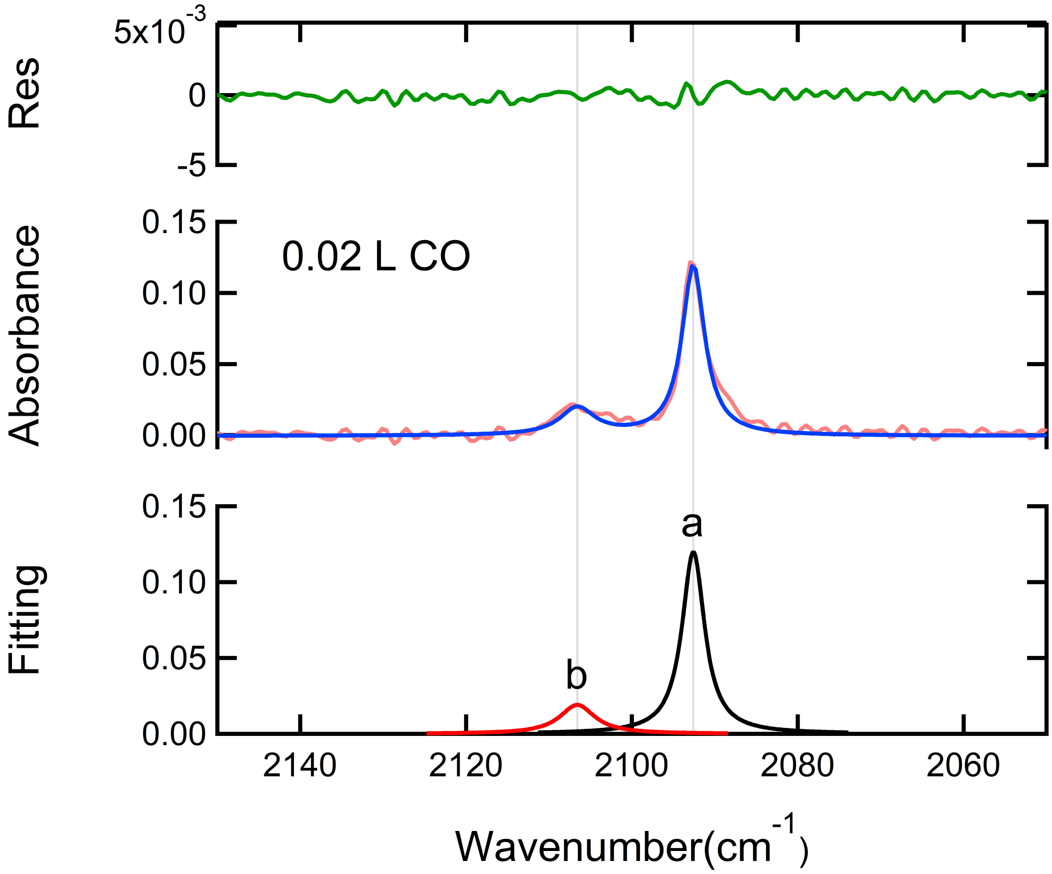

Figure S2: Fitting of absorbed CO RAIR spectra on clean Cu(211) with a combination of two independent asymmetric pseudo-Voigt profiles for an exposure to 0.02 L CO (center panel) with residuals (top panel) and separation of the two features (bottom panel).

In Figure S2, we exemplify the fitting of absorbance bands in our RAIR spectra. We show the fit to the data for on-top bound CO on clean Cu(211) at an exposure of 0.02 L CO. The pink trace in the center panel shows the experimental data. The blue line in the same panel is the best fit that combines two independent asymmetric pseudo-Voigt profiles.[3] The lower panel shows the two absorbances with color coding: black (label a) for the absorbance near 2095 cm^{-1} and red (label b) for the absorbance near 2109 cm^{-1}. The upper panel shows the residuals of the fit.

Important fit parameters that we obtain from all available CO spectra and clean Cu(211) are plotted as a function of CO exposure in Figure S3. A selection of the spectra is shown

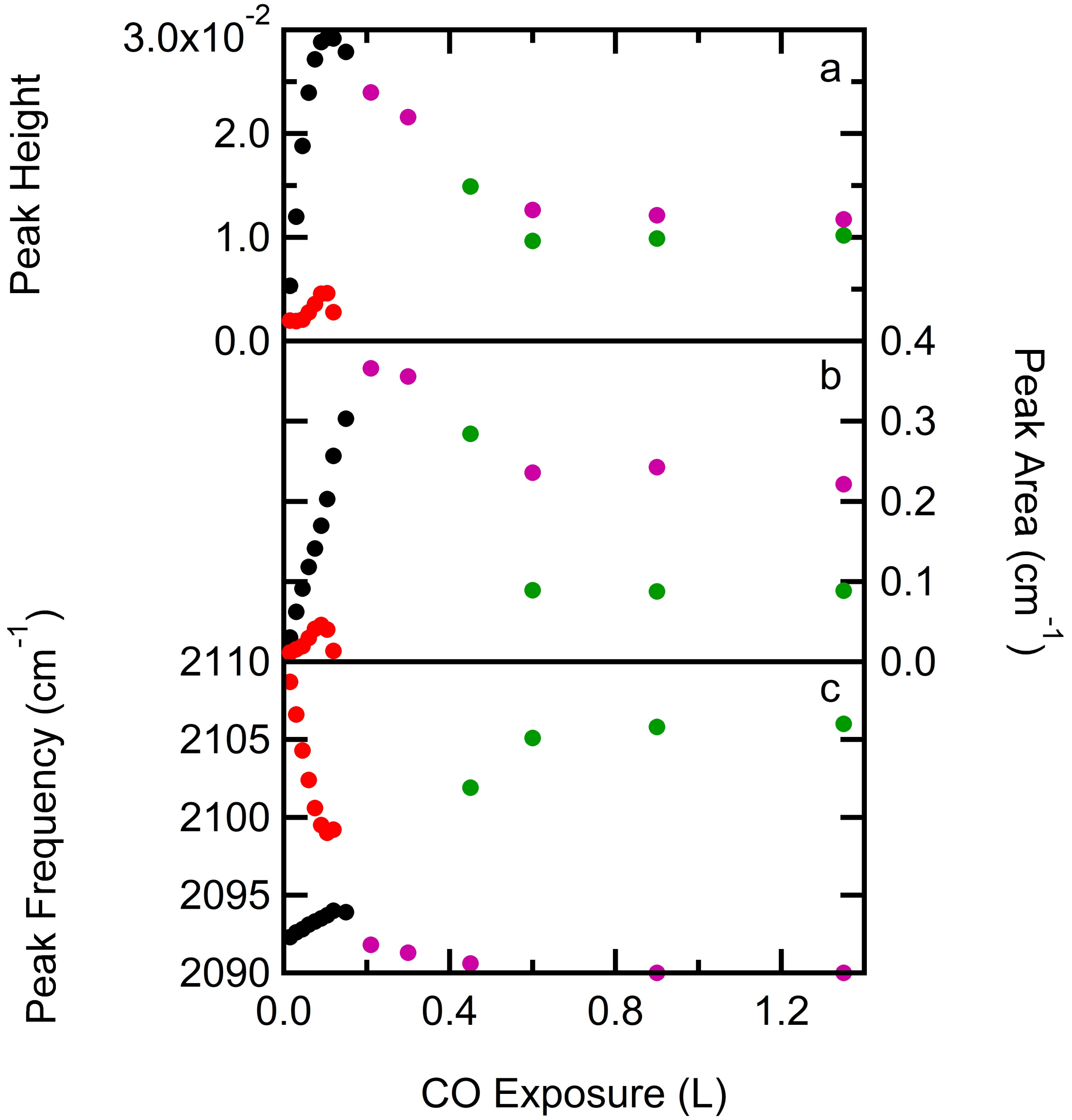

Figure S3: Development of three characteristic values resulting from CO IR adsorption profile fits as a function of CO exposure for three different peaks onto metallic Cu(211). a) peak height, b) peak area, and c) peak frequency. The parameters result from pseudo-Voigt profile fitting to IR spectra such as shown in Figure S2. The black and red colored data represent the same peaks as shown in Figure S2, i.e., the modes resulting from kink and step occupancy. In purple, we show the fit parameters starting when the kink absorbance has fully disappeared. The green data apply to the higher frequency mode when two absorbances are clearly present at the higher CO exposures.

in Figure 5a. The red and black markers reflect the two absorptions also shown in Figure S2 as red and black, and which develop with CO dose as shown in Figure 5a. The green and purple markers reflect the fit parameters for the absorbance(s) observed at CO doses above 0.2 L. The maximum absorbance (peak height) is shown in the top panel. From the fitted function, we calculate the integrated band intensities (”peak area”). These are reported in the center panel. The frequency of maximum absorbance (ν_p or peak frequency) is shown in the bottom panel.

The data in Figure S3 show the evolution of the two absorptions with singleton frequencies 2093 (black markers) and 2109 cm^{-1} (red markers) extrapolated to zero coverage. The same peaks were observed by Pritchard and Hollins for low doses[4] and on our previously used, but slightly contaminated, Cu(211) crystal (see Figure S9). Considering the surface structure and very low CO dose, the signals may be assigned to occupancy of a small number of kink sites (2109 cm^{-1}) and a much larger number of step sites (2093 cm^{-1}). The former saturates first, as expected, since kink-like isolated Cu atoms are known to bind CO more strongly than step edges.[5]

With increasing CO dose, the separation between the center frequencies decreases until, at 0.1 L CO, the two peaks have effectively merged. The peak height, band intensity, and frequency shift for the dominant mode (black markers) are clearly linear with CO dose in this range. Once the kink sites are saturated, their signal is overwhelmed by the still-increasing coverage of step sites. This qualitative change in the spectrum is consistent with expectations for a minority species resonantly coupled to the majority, at constant local density but increasing overall dilution.[6]

When the CO dose reaches 0.11 L, its spectrum is dominated by a single symmetric peak assigned to occupied step sites. We find no evidence of bridge site adsorption at any coverage, as shown in Figure S4. Here, spectra for the atop and bridge adsorption ranges for CO/Cu(211) are shown side by side. Theoretical studies of CO adsorption to bridge sites of Cu(211) report vibrational frequencies in the range 1850-1950 cm^{-1}. Because the

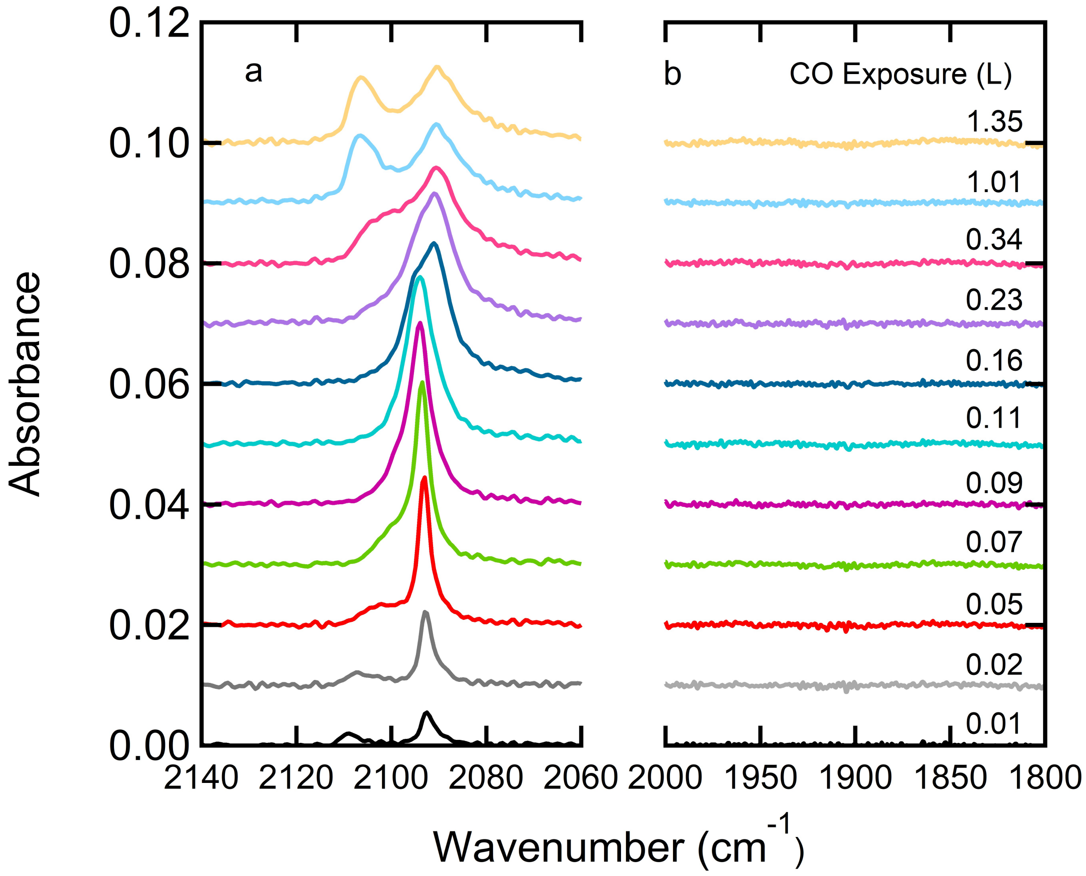

Figure S4: IR absorbance spectra over frequency ranges covering a) atop and b) bridge adsorption of CO on clean, metallic Cu(211) for various exposures indicated near the traces.

infrared cross-section at bridge sites is generally weaker than at atop sites, our observations do not rule out the proposed occupancy of bridge sites in a (1×3) periodicity at lower surface temperatures.[5,7] Both the higher mobility of CO at our higher surface temperature, and the considerably weaker absorbance may contribute to the absence of the typical IR signature near 1850 cm^{-1}. That said, bridge-adsorbed CO is clearly discernable in our spectra of the flat Cu(111) surface, shown in Figure S7. So the single symmetric peak at intermediate exposure represents parallel rows of step-adsorbed molecules, with the occasional kink, separated by largely empty terraces. If isolated terrace-bound molecules are present, their oscillator strength will be transferred to the high-frequency in-phase mode.

The final splitting of the adsorbed CO peak is first visible as an unresolved doublet at 0.16 L and is clear above a ~0.3 L CO dose. Since neither component is bridge-bound, we propose that the new lower-frequency signal arises from molecules adsorbed atop terrace atoms of local (111) character. These rows of molecules will therefore resemble CO adsorption to Cu(111) itself. At high coverage, the latter appears at 2069-2074 cm^{-1} (see our previous study,[8] Figure 4 of this manuscript, and RAIR spectra of saturated CO overlayers on Cu(111) in Figure S7).

Exposures beyond step saturation increase the density of molecules within the terrace-adsorbed rows. The separation between singleton frequencies at edges and terraces is small enough (~5 cm^{-1}) that dipole-dipole coupling will affect the final disposition of peak frequencies (increased separation) and intensities (transfer to the high-frequency mode).[6] The observed shift of the high-frequency in-phase mode to higher frequency is indeed observed. The final, well-resolved doublet at 2090 and 2106 cm^{-1} thus arises from interdigitated rows of edge- and terrace-bound CO. However, the low-frequency out-of-phase mode remains unexpectedly intense.[6] Quantitative support for the coupling scenario described here will require bandshape simulations that include both the anisotropy of coupling within the layer and sequential filling of the site types. Additionally, since the TPD peak assigned to terrace-bound molecules is also larger than that for the step-edge sites (Figure 6), it is possible that

the saturation coverage of terrace sites is in fact greater than that of step-edge sites.

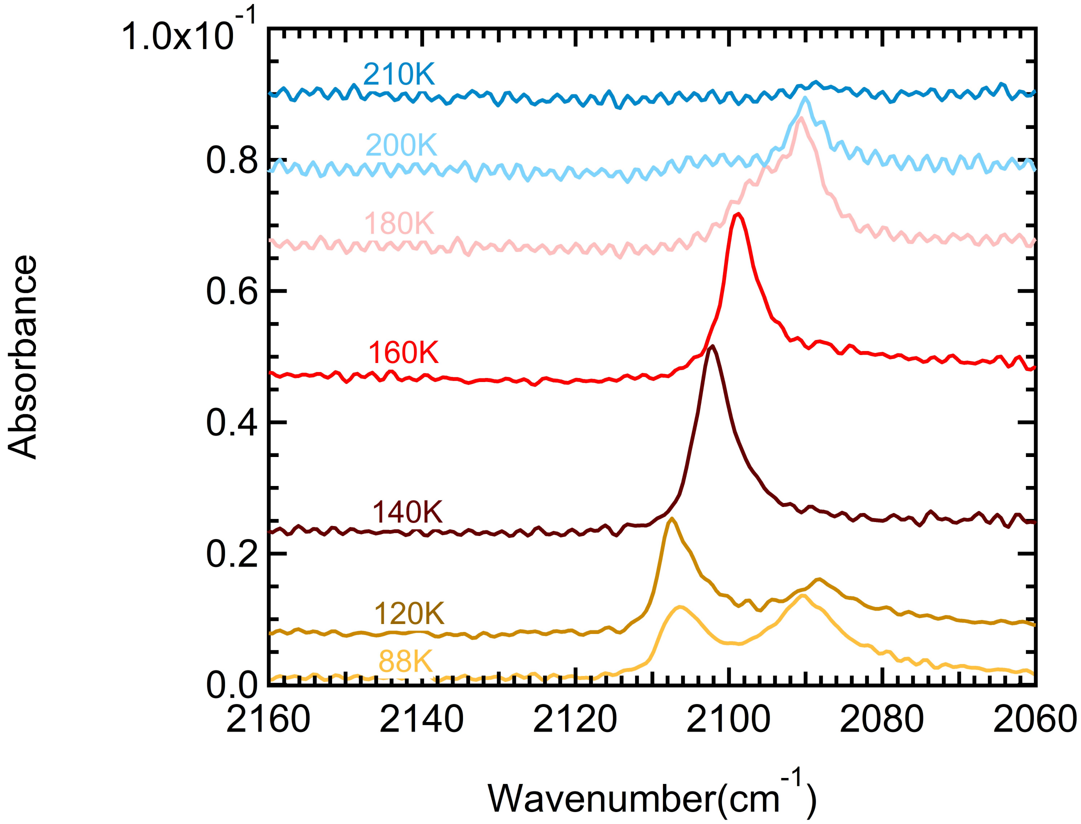

Figure S5: IR absorbance spectra of a 1.35 L CO dosed onto clean Cu(211) at 88 K with subsequent increases in surface temperature. The increasing oscillations in the background result from the IR emission of the filament that heats the Cu sample.

As an additional test of the preceding interpretation, we consider how the IR spectrum evolves as the adsorbed layer evaporates. Figure S5 shows spectra obtained at increasing temperatures, beginning with a saturated CO overlayer at 88 K (bottom trace) which is comparable to the saturated doublet in Figures 5a and S3. 120 K is the onset of terrace desorption in TPD measurements and, accordingly, the low-frequency member of the IR absorption doublet is diminished. In the range 140-160 K, where the rate of terrace desorption is the greatest, the corresponding IR signal has been removed entirely. The higher-frequency mode shifts to the red, as the extent of dynamic dipole-dipole coupling decreases with decreased local density of the adsorbed layer. 180 K marks the onset of desorption from step edge sites. As the rows of step-bound molecules disintegrate, their vibrational frequency

returns to the singleton value near 2095 cm^{-1}. A high-frequency shoulder representing CO strongly bound to kink defects persists to 200 K, above which the surface appears clean in the time it takes to record a spectrum. The final signals appear at $\sim$5 cm^{-1} higher frequency than those seen at the lowest initial exposure (Figure 5a); the shift may be caused by a different sampling of surface heterogeneities or the different temperature at which the spectra were recorded.

RAIR spectra fitting results for $CO/Cu_xO/Cu(211)$

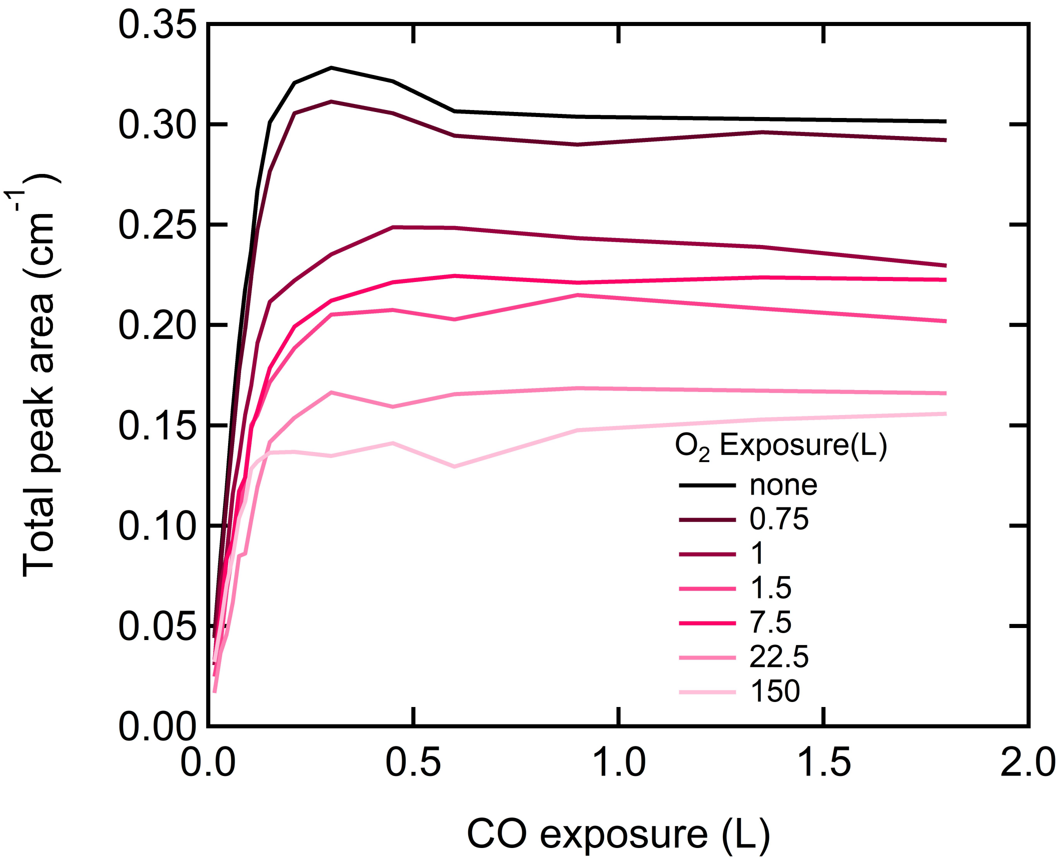

Figure S6: Relative spectral absorbance peak area of CO exposure on clean and O pre-covered Cu(211). The O_2 pre-dosage is indicated in the legend.

The total CO absorbance plotted in the center panel of Figure S3 has also been determined for the case of Cu(211) pre-exposed to O_2. Figure S6 shows the absorbance as a function of CO exposure for six different O_2 pre-exposures and for the clean Cu(211) surface. All cases behave similarly. Over the range of 0 to ∼0.12 L of CO exposure, the total absorbance increases linearly with CO exposure before slowing down and reaching a plateau at ∼0.3 L. The level of the plateau decreases with increasing O_2 pre-exposure.

A comparison of (Cu_2O/)Cu(111) prepared with thermal and supersonic O_2 sources

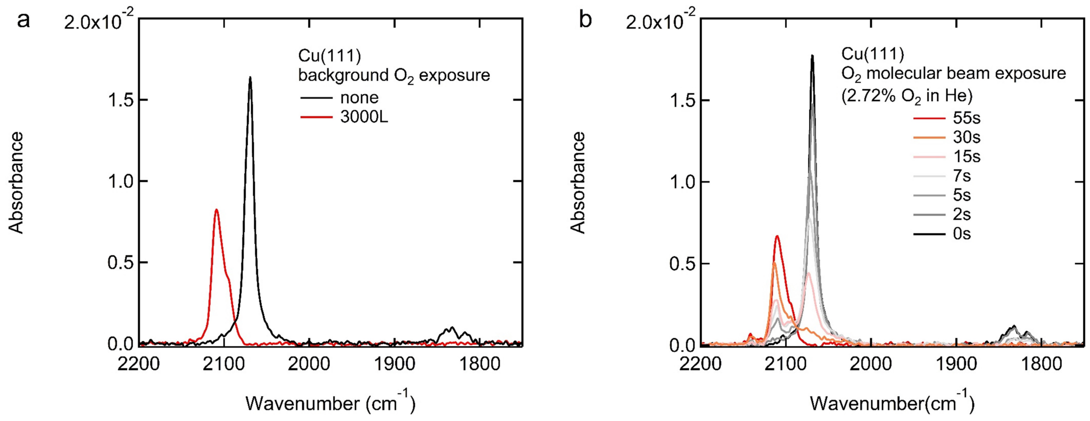

Figure S7: CO absorbance comparison for Cu(111) with oxidation by O_2 using a) background-dosing and b) supersonic molecular beam dosing with O_2/He.

Figure S7 compares IR spectra of CO adsorbed to Cu(111) obtained after a) background dosing of O_2 and b) supersonic molecular dosing. The clean Cu(111) surface was subsequently exposed to approximately 0.68 L CO by background dosing (black trace in figure S5a). This dose nearly saturates the surface. The spectrum reproduces the previously found absorbances at 2069 cm^{-1} for CO adsorbed on top sites and the doublet at 1817 and 1833 cm^{-1} for CO adsorbed on bridge sites.[9] After a very large exposure to O_2 at room temperature, i.e. 3000 L (red trace), there is no more evidence of bridge-bound CO and the top-site absorbance has shifted to higher frequencies. When the measurement is repeated with a helium-seeded supersonic molecular beam as the source of O_2, nearly identical spectra are obtained, as shown in Figure S7b. Both top and bridge-site absorptions gradually disappear with molecular beam doses between 0 and 55 s. Beyond a 55 s dose, no further dissociative O_2 adsorption is observed, indicating completion of the stoichiometric Cu_2O surface. The only difference between the two exposure methods is an additional weak absorption at 2142 cm^{-1} in the molecular-beam-dosed O_2 case. This comparison testifies that the average kinetic energy of the O_2 impinging onto Cu(111) at room temperature does not strongly affect the

ultimate oxide surface structure, as probed here by post-adsorbing CO.

Integrated CO TPD spectra

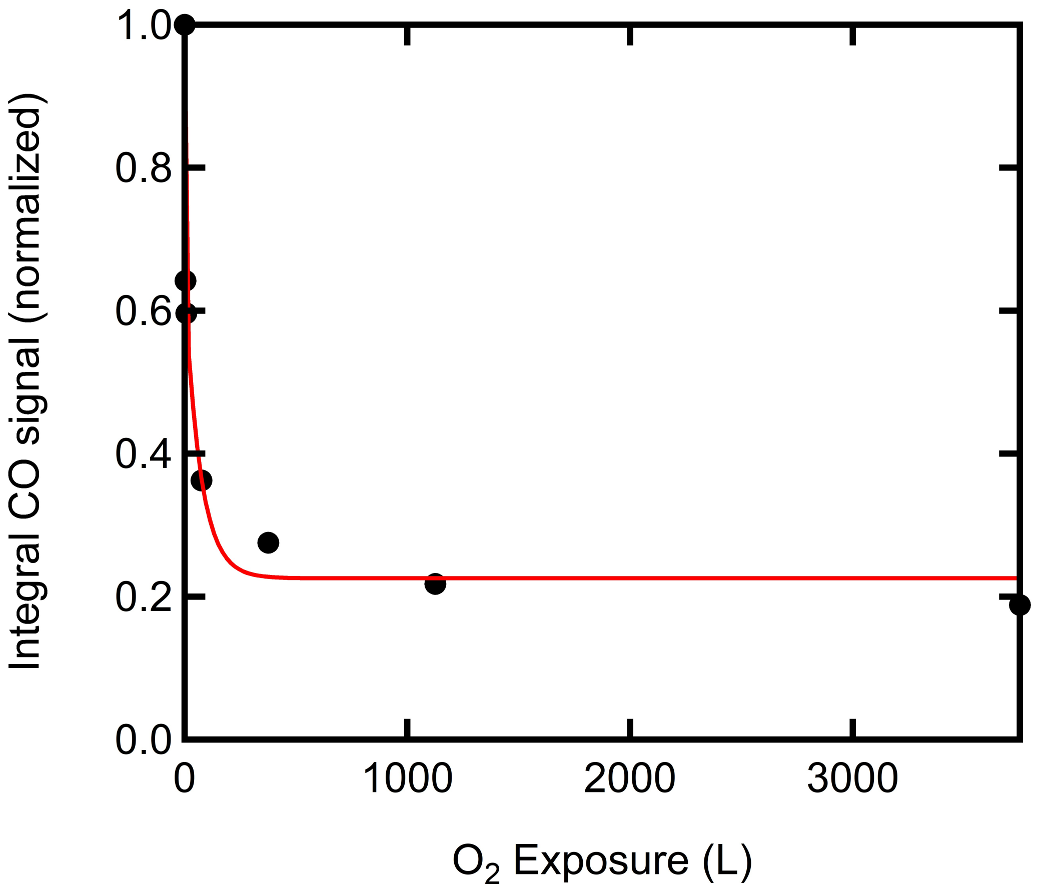

Figure S8: Integrated CO TPD spectra from a 0.23 L CO dose at <100 K vs prior O_2 dose at 300 K on Cu(211) with an intermediate flash to 450 K. The red solid line is a double exponential function fit serving to guide the eye.

Figure S8 shows integrated and normalized TPD signals versus the O_2 exposure for Cu(211). While low levels of oxidation rapidly decrease the attained CO coverage for a fixed CO dose (i.e. 0.23 L), oxidation beyond O_2 exposures of several tens of Langmuirs do not affect the CO coverage further. It settles at approximately 20% of the initially attained CO coverage on clean Cu(211).

RAIR spectra for CO adsorbed to a weakly contaminated Cu(211) single crystal

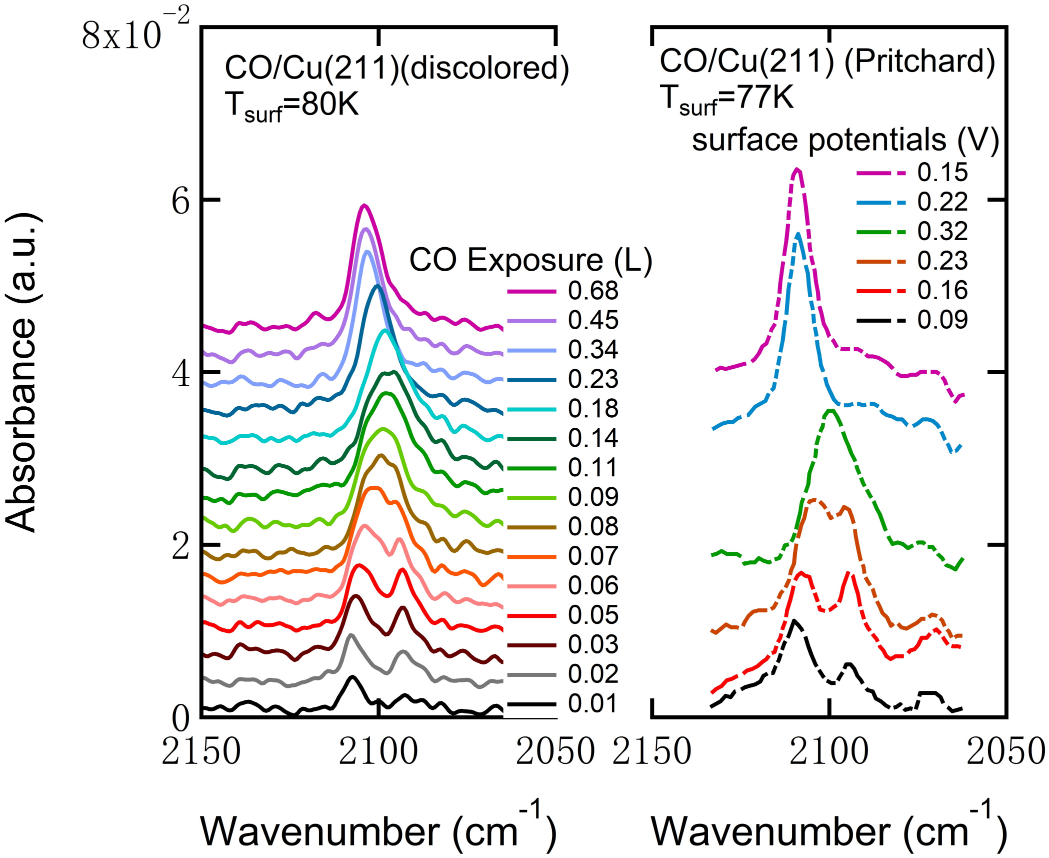

Figure S9: Comparison of RAIR spectra of increasing CO doses onto our first used Cu(211) single-crystal surface and results from Pritchard et al.[4]

Our initial experiments in this study used a Cu(211) single crystal that was most likely contaminated by small amounts of Rh in the course of experiments. A hot Rh filament was held in front of the Cu(211) surface, in a background of O_2 gas, in an attempt to dose O atoms onto the cooled surface prior to exposing it to CO. This was done to co-create O_{ads} and CO_{ads} at a low surface temperature under the assumption that it may keep the two adsorbates more closely spaced. We noticed a discoloration of the Cu(211) surface after several weeks of experiments. A band of silver-white metallic color appeared in the center of the crystal face, where the Rh filament was closest. We have been unable to detect the

suspected Rh or other contaminants by AES, suggesting that the contamination level is low. However, it clearly affects the reflective optical properties of the Cu surface.

RAIRS spectra taken after exposing the contaminated surface at 90 K to various doses of CO are shown in Figure S9. They mostly match the results obtained later on a new and clean Cu(211) surface as shown in Figure 5a. They also match CO spectra by Pritchard and Hollins,[4] included for comparison. Pritchard and Hollins report the associated surface potentials as detected by a Kelvin probe instead of CO exposures. The notable differences between these spectra and our later measurements from a clean Cu(211) surface are the slightly stronger relative absorbance by kink defects around 2110 cm^{-1} for the lowest CO exposures, the broader absorbance of the main feature around 2095 cm^{-1} at intermediate exposures, and the absence of splitting of this main feature at the highest exposures. This comparison underlines that subtle contamination—undetectable by AES—may have effects on RAIR spectra of adsorbed CO.

References

(1) Witte, G.; Braun, J.; Nowack, D.; Bartels, L.; Neu, B.; Meyer, G. Oxygen-induced reconstructions on Cu (211). *Physical Review B* **1998**, *58*, 13224.

(2) Thompson, K.; Fadley, C. X-ray photoelectron diffraction study of oxygen adsorption on the stepped copper surfaces (410) and (211). *Surface science* **1984**, *146*, 281–308.

(3) Stancik, A. L.; Brauns, E. B. A simple asymmetric lineshape for fitting infrared absorption spectra. *Vibrational Spectroscopy* **2008**, *47*, 66–69.

(4) Pritchard, J.; Catterick, T.; Gupta, R. Infrared spectroscopy of chemisorbed carbon monoxide on copper. *Surface Science* **1975**, *53*, 1–20.

(5) Gajdoš, M.; Eichler, A.; Hafner, J.; Meyer, G.; Rieder, K.-H. CO adsorption on a Cu

(211) surface: First-principle calculation and STM study. *Physical Review B* **2005**, *71*, 035402.

(6) Hollins, P. The influence of surface defects on the infrared spectra of adsorbed species. *Surface Science Reports* **1992**, *16*, 51–94.

(7) Meyer, G.; Rieder, K., et al. Identification of ordered CO structures on Cu (211) using low temperature scanning tunneling microscopy. *Chemical physics letters* **1995**, *240*, 379–384.

(8) Zhang, D.; Jansen, C.; Berg, O. T.; Bakker, J. M.; Meyer, J.; Kleyn, A. W.; Juurlink, L. B. RAIRS Characterization of CO and O Coadsorption on Cu (111). *The Journal of Physical Chemistry C* **2022**, *126*, 13114–13121.

(9) Raval, R.; Parker, S.; Pemble, M.; Hollins, P.; Pritchard, J.; Chesters, M. FT-rairs, eels and leed studies of the adsorption of carbon monoxide on Cu (111). *Surface Science* **1988**, *203*, 353–377.
